# Prevalence and economic losses of calf fetal wastage in ELAKAT public slaughterhouse of Bukavu, Democratic Republic of Congo

**DOI:** 10.14202/vetworld.2019.1644-1649

**Published:** 2019-10-26

**Authors:** Valence Bwana Mutwedu, Bernard Kanyungu Buuma, Arsène Ciza Mushagalusa, Ntagereka Patrick Bisimwa, Nadège Cizungu Cirezi, Yannick Mugumaarhahama, Rodrigue Balthazar Basengere Ayagirwe

**Affiliations:** Department of Animal Production, Faculty of Agricultural and Environmental Sciences, Université Evangélique en Afrique, Bukavu, Democratic Republic of the Congo

**Keywords:** cattle, Democratic Republic of Congo, economic implications, fetal loss, South Kivu

## Abstract

**Aim:**

This study was conducted to assess the prevalence of calf fetal wastage and its economic implications at ELAKAT slaughterhouse, Bukavu, Democratic Republic of Congo (DR Congo) to fill the research gap in relationship with this thematic. The study investigations took place from May to September 2018.

**Materials and Methods:**

A cross-sectional survey was carried at the slaughterhouse. For each visit, the number of cattle slaughtered, the number of pregnant cows slaughtered, and gestational ages (stage of pregnancy of the dam, and estimated by crown-rump length) of the fetuses were recorded.

**Results:**

Out of the 1035 cattle slaughtered during the study period, 970 were females. A total of 255 fetuses were recovered, representing a fetal recovery rate of 26.28%. The study established that one fetus was lost out of 4.5 cows slaughtered, and most of fetuses recovered (58.1%) were in the first trimester of gestation while 29.1% and 12.8% were, respectively, in the second and third trimester. Their age varied from 1.2 to 8.6 months, with body length ranging from 14 cm to 92 cm while their weight varied from 1.0 kg to 23.0 kg. The economic loss associated with the total cattle fetal wastages was estimated at Congo Democratic Francs (CDF) 29,906,400 ($15,787.5) with a monthly average of CDF 5,981,280 ($3,157.5).

**Conclusion:**

These results attested that slaughtering pregnant cows constitute a strong constraint on cattle industry development in DR Congo. Urgent measures, such as adequate enforcement of legislations on routine veterinary examinations at slaughterhouses as well as livestock owner’s sensitization, are required to avoid selling pregnant cows during calving season.

## Introduction

Animal protein consumption is very important in body defense mechanisms due to its better balance of amino acids [1]. These proteins are mainly provided by cattle for human population [2]. In Africa, cattle are of crucial importance through their contribution to the improvement of nutritional status and the economic growth of their owners [3]. As reported in many African countries, cattle are mainly reared for meat and milk production. They also provide job opportunities and represent an important source of income and energy, as well as skin, hair, hide products, and manure as by-product [4,5].

However, in Africa, the annual consumption of animal protein remains very low compared to what is reported in Australia and the USA (approximately 115.5 kg/person/year) and even low compared to the global average of 41.9 kg/person [6].

In the Democratic Republic of Congo (DR Congo), more specifically, livestock contributes up to 9.2% of the gross domestic product and plays a crucial role in the livelihood of the local population. Cattle, which is the most reared livestock and the most consumed in the country, contributes to more than 50% of the total meat consumption [7].

The DR Congo cattle herd size is about 1,005,385 heads representing 11% of overall livestock population [7], while the pastoral potential is estimated between 30 and 40 million heads showing how much protein availability is a serious challenge [8].

This low cattle productivity observed in DR Congo as in other African countries is constrained by several factors such as disease, inadequate nutrition, poor management, and low biosecurity practices, with little or no veterinary attention, low genetic potential of native livestock, lack of concrete national policies, low quality feeds and higher costs of commercial feed, insecurity in rural regions, and reproductive wastages [4,9-12]. All these factors have constrained livestock production to remain at a subsistence level [11].

In addition, due to the steady animal protein demand accentuated by population growth, illiteracy, poverty of farmers, and disease condition of animals, farmers sell off animals without considering their fertility stage, especially breeding stock and pregnant animals followed by inadequate meat inspection practices [13-17]. Thus, fetuses are usually discovered during postmortem meat inspection that may have negative effect on livestock growth capacity, giving poor meat quality to consumers, and represent a serious problem for animal ethics [18,19]. This leads toward not only wastage of scarce protein by supplying poor quality meat products to consumers, but also a decrease in livestock growth capacity at country level as well as low herd replacement rates [11].

Wastage of fetuses through the indiscriminate slaughter of pregnant female animals is one of the most destructive practices humankind has ever used against his production endeavor. Lack of legislation strictly regulating abattoir construction and operations, the unavailability of modern abattoir where proper antemortem examinations of slaughtered animals are practiced to eliminate slaughtering of pregnant cows remains the factor of this practice spreading [11,14].

In Africa, calf losses associated with the slaughter of pregnant cows are enormous and have been documented in several countries such as Nigeria [14,20-22], Cameroon [23,24], Tanzania [25,26], and Ghana [19], but there is a dearth of similar investigations in DR Congo.

Despite the concern raised on this thematic in other countries, no study has been carried out in DR Congo particularly in South Kivu to investigate the empirical evidence of the fetal wastages incidence through slaughter of pregnant cattle and their effect on public health, livestock production, national herd size, and economic implication to better planning, and implement policies on meat inspection.

Therefore, this study was designed to investigate the incidence of calves’ fetal wastages at ELEKAT public slaughterhouse of Bukavu city and estimate its economic implications.

## Materials and Methods

### Ethical approval

The study protocol was approved by the Ethical Committee of the Université Evangélique en Afrique (UEA).

### Study area

This study was carried out at ELAKAT public slaughterhouse of Bukavu in the South Kivu province, Eastern DR Congo.

ELAKAT slaughterhouse is the main place where most of the cattle are slaughtered for meat supply in Bukavu and other neighboring cities. It is the largest abattoir in Bukavu established during the colonial period where the population was estimated at 805,550 in 2017 [27].

ELAKAT is located in the South-Eastern part of Bukavu in a small basin of Ruzizi River catchment at the border between DR Congo and Rwanda. Moreover, the place constitutes the main entry point for imported cattle in town and within the district. Cattle are slaughtered on a daily basis with an average number of 20-50 cattle slaughtered depending on days, weeks, and months [28].

### Data collection

From May to September 2018, records of calf fetal wastages resulting from slaughtered pregnant cows were collected daily (except Sunday) between 8:00 am and 11:00 am under the supervision of a provincial veterinarian. Before animal slaughtering, the sex (by exploring external genital organs) of each animal was recorded, as well as counting and tallying at the main entrance, as animals were led into the slaughter point. The pregnancy status of the cows was first determined by visual assessment and palpation of the exposed uterus after slaughtering and then confirmed by dissecting the uterus of any suspected gravid cow. Fetuses’ sex was determined by exploring external genital organs. On the other hand, weight was determined using a mechanical weighing scale while the age was estimated based on the fetus crown-rump length using the formula as suggested by Singh *et al*. [29]:

Y = 24.42 + 0.39X where:

Y = Gestational age of the fetus.

X = Crown-rump length in millimeters.

The age of fetuses allowed to classify three different gestation stage types (first, second, and third trimesters, when the calculated age, respectively, fell between 1 and 3 months, 4 and 6 months, and 7 and 9 months).

The economic losses from the calf fetal wastage were determined according to the method developed by Alhaji *et al*. [14]; a kilogram of cattle live weight estimated at Congo Democratic Francs (CDF) 4800 ($3) (source: Butchers and local population).

### Statistical analysis

The data obtained were analyzed using descriptive analysis, such as simple averages and percentages. The prevalence of pregnant cows slaughtered was determined as the proportion of the total number of slaughtered females that were pregnant at the time of slaughter. The prevalence of fetal wastage on the other side was determined by dividing the number of wasted fetuses by the number of slaughtered females and also expressed the risk of fetal wastage associated with slaughtering any pregnant or non-pregnant female.

## Results

### Incidence of fetal wastage at ELAKAT provincial slaughterhouse

During the investigation, a total of 1035 cattle were slaughtered. The number of slaughtered females was 970; among them, 255 were pregnant, representing 26.3% of the cows slaughtered, resulting in one fetus lost after 4.5 cows slaughtered. The monthly average of 51 wasted fetuses was recorded for 194 cows slaughtered (Table-1).

**Table 1:** Incidence of fetal wastage at ELAKAT provincial slaughterhouse.

Month	Total cattle slaughtered	Cow slaughtered	Non-pregnant cows slaughtered	Pregnant cow slaughtered	Rate of calf fetal wastage (%)	Slaughtered cow/pregnant cow ratio
May	241	230 (95.4)	166 (72.2)	64 (27.8)	27.8	3.6
June	196	185 (94.4)	131 (70.8)	54 (29.2)	29.2	3.4
July	210	199 (94.8)	144 (72.4)	55 (28.6)	27.6	3.6
August	244	238 (97.5)	173 (72.7)	65 (27.3)	27.3	3.7
September	144	118 (81.9)	101 (85.6)	17 (14.4)	14.4	8.4
Mean	207	194 (93.7)	143 (73.7)	51 (26.3)	26.2	4.5
Total	1035	970 (93.7)	715 (73.5)	255 (26.5)	26.3	4.4

Values in bracket represent fetal wastage proportion (%)

### Monthly summary of wasted at ELAKAT provincial slaughterhouse

From Table-2, it appears that independent of the month; female fetuses (59.2%) were most lost compared to males (40.8%). Their ages varied from 1.2 to 8.6 months, with body length ranging from 14 cm to 92 cm. Fetus weight varied from 1.0 kg to 23.0 kg, with an average weight of 3.6 kg.

**Table 2:** Characteristics of fetuses wasted at ELAKAT provincial slaughterhouse.

Parameters	Modality	May	June	July	August	September	Mean
Sex	Male	29 (45.3)	17 (31.5)	18 (32.7)	29 (44.6)	11 (64.7)	20.5 (40.8)
	Female	35 (54.7)	37 (68.5)	37 (67.3)	36 (55.4)	6 (35.3)	30.2 (59.2)
Age (month)	Minimum	1.2	2.0	1.8	1.6	1.2	1.5
	Mean	3.9	4.4	3.9	3.9	4.3	4.1
	Maximum	8.4	8.6	8.0	7.7	7.9	8.2
Length (cm)	Minimum	14.0	18.0	17.0	16.0	14.0	15.5
	Mean	26.1	30.9	26.2	25.3	29.4	27.3
	Maximum	89.0	92.0	80.0	75.0	78.0	84.3
Weight	Minimum	1.0	1.0	1.0	1.0	1.0	1.0
	Mean	3.5	4.3	3.8	2.9	3.6	3.6
	Maximum	23.0	23.0	22.0	18.0	20.0	21.5

Values in bracket represent fetal wastage proportion (%)

### Pregnancy status of cows slaughtered at ELAKAT provincial slaughterhouse

Table-3 indicates that 58.0% of the slaughtered cows (148 cows) were in their 1^st^ trimester (0-3 months), 29.1% (74 cows) were in their 2^nd^ trimester (4-6 months), and only 12.9% (33 cows) were in their 3^rd^ trimester (7-9 months).

**Table 3:** Pregnancy status of the female cows slaughtered at the ELAKAT provincial slaughterhouse.

Month	Trimesters			Total

	1^st^ trimester	2^nd^ trimester	3^rd^ trimester	
May	35 (55.7)	21 (32.8)	8 (11.5)	64
June	30 (54.8)	12 (22.6)	12 (22.6)	54
July	32 (59.3)	16 (29.6)	6 (11.1)	55
August	35 (57.4)	22 (33.3)	8 (9.3)	65
September	13 (66.7)	3 (76.4)	1 (5.9)	17
Mean	29.6 (58.1)	14.8 (29.1)	6.5 (12.8)	51
Total	148 (58.0)	74 (29.1)	33 (12.9)	255

Values in bracket represent fetal wastage proportion (%)

### Financial implications of slaughtering pregnant cows at ELAKAT provincial slaughterhouse

The calculated values for losses due to fetal wastage as a result of slaughtering pregnant cows are shown in Table-4. The average monthly economic loss associated with slaughtering pregnant cows in this study is estimated at CDF 5,981,280 ($3,157.5). During the 5 months of study, the total economic losses can be estimated at CFD 29,906,400 ($15,787.5).

**Table 4:** Average monthly financial implications of slaughtering pregnant female cows at the ELAKAT provincial slaughterhouse.

Month	Number of fetuses wasted	Congo Democratic Franc	Amount loss (USD)
May	64	6,528,000	4896
June	54	6,609,600	4131
July	55	6,732,000	4207.5
August	65	7,956,000	4972.5
September	17	2,080,800	1300.5
Mean	51	5,981,280	3157.5
Total	255	29,906,400	15,787.5

## Discussion

The results of this study indicate that more cows have been slaughtered than bulls. These findings are in accordance with those observed by researchers in other cattle slaughterhouses [14,21,30,31]. This represents huge losses to the reproductive efficiency and propagation of livestock populations, as many more females are required for reproduction purposes than males. Slaughtering females are, therefore, to be prohibited for animal production and should be done with discretion [32]. Riehn *et al*. [33] indicated that economic livestock management demands that animals sold for slaughter should be mainly males and reproductively inactive females.

The decrease in the number of slaughtered cattle in September could be associated with the dry season. Indeed, the research zone covered by this study has two seasons, namely, the rainy season (September to mid-May) and the dry season which lasts from May to mid-September. During the dry season, most farmers prefer reducing their animal flock size as feed availability poses problems during this period.

The average prevalence of slaughtering pregnant cows (26.29%) reported in this study is around those reported 22.4% in Jos abattoir of Nigeria [20], 22.10% in Bamenda slaughterhouse of Cameroon [23], 24.06% in Sokoto abattoir of Nigeria [34], and 26% in Enugu abattoir of Nigeria [35]. The high prevalence of pregnant cows slaughtered in this study could be due to the higher number of cattle slaughtered, this is proportional to the number of cows and cattle slaughtered and are expected to have effects on fetal wastage [3]. In addition, with the increase of farming activities within the study area, farmers sell even pregnant cows as they need more financial resources for the purchase of farm inputs like seeds, fertilizer and herbicides as highlighted in earlier studies[11].

The results of this study indicate that one fetus was lost each time 4.5 cows were slaughtered. This result agrees with the findings in Bamenda slaughterhouse of Cameron (one fetus for every four cows slaughtered) [23] but higher than the findings in Lafenwa abattoir in Nigeria (one fetus for every seven cows slaughtered) [3], at Gombe abattoir in Nigeria reported one fetus lost for every 33 cows slaughtered [36] while at Jos abattoir in Nigeria recorded one fetus for every eleven cows slaughtered [37]. This higher fetal wastage rate reported in this study may be due to the slaughtering of pregnant animals for the purposes of festivals, ceremonies, disease, and poverty [3]. Financial resources’ limitation in the time of crisis such as dry season may motivate indiscriminate sale of females for slaughter [25] as well as uncontrolled reproduction system do not allow to discern pregnant animals as any diagnostic test is done at farm level. This high fetal wastage rate is quite alarming, and effort should be geared toward instituting routine veterinary checks including pregnancy diagnosis at cattle control posts and abattoirs. However, many efforts should be put at increasing future domestic meat supply to reduce the incidence of slaughtering pregnant cows [19].

Results from Table-2 indicate that most of the pregnant cattle slaughtered are at the 1^st^ pregnancy trimester (58%). This is not surprising, as in the first trimester it is very difficult to notice the gestation than at the second and third trimesters where it can be readily detected. Swai *et al*. [25] indicated that, in Africa, the reason for slaughtering pregnant animals is that pregnancy diagnoses are not routinely conducted during antemortem inspection in the abattoir due to various reasons including poor infrastructures and staff competency in carrying out pregnancy diagnosis. These findings are in discordance to those found at Nsukka slaughterhouse [11], in North-Eastern Nigeria [13], in Makurdi abattoir [22] reported that major cows’ pregnancies were at the 2^nd^ and 3^rd^ trimesters. The general reason for the slaughter of pregnant cows at these trimesters could be attributed to the high demand and preference for big-sized cows. As a result of poverty, ignorance, and illiteracy of the livestock owners, they dispose of the supposedly big cattle that are weighty and with shiny hair coat because they fetch a higher price, to meet their household needs which include payment of school fees, festivals, ceremonies, and agricultural activities [25,26]. Since no antemortem inspection is carried out in ELAKAT slaughterhouse, the proportion of fetal wastages observed in this study accounts for a considerable loss of animal protein and future national herd.

The financial implications of fetal wastage in this study indicate economic and production losses. In fact, the results of our findings revealed that a total of 255 fetuses were wasted within the 5-month study duration. This shows that, in just 5 months, there is a reduction of 255 cows from the ELAKAT slaughterhouse. However, when considering a birth weight of 25.5 kg and market price of CDF 4800 ($3)/kg live weight, if calves were slaughtered immediately after consuming colostrum at birth, the financial loss due to fetal wastage during the 5-month study will be 29,906,400 Congolese Francs or $15,787.5 American dollars (Central Bank of DR Congo’s exchange rate, 2018). These results are low considering the findings of Dunka *et al*. [20] during 60 months ($571,830.07) and Alhaji [14] during 108 months ($56,828.57). However, the results of this study, which only covers 5 months, are alarming when compared to the investigations period of the above-mentioned studies. As augured by FAO, this loss of cattle production, if not solved, is likely to affect the security of future sources of animal protein considering the increase in human population [38].

These results indicate that, if considered nationwide, the slaughtering of pregnant food animals has serious economic and food security implications on the Congolese livestock economy.

## Conclusion

The results of these findings testify that the prevalence of calf fetal wastage and the economic implications resulting from the slaughter of pregnant cows at ELAKAT public slaughterhouse of Bukavu city, DR Congo, are enormous, and this seems to continue as much as the demand and consumption of cattle meat is increasing. Thus, measures must be taken in agreement with all actors within the meat value chain such as breeders, animal traders, and inspection officers affected to the slaughterhouses to reduce this extent. It would be necessary to set up early pregnancy diagnosis techniques in slaughterhouses, to train and retrain inspectors and slaughter agents so that a pregnancy diagnosis can be made, even at a very early stage. Besides, it is noteworthy to ensure the establishment and enforcement of animal slaughter legislation which prohibits the slaughtering of pregnant animals with the exception of the animals being culled.

## Authors’ Contributions

VBM and RBBA made contribution to the conception, design, provision of field sample, and drafting the manuscript, BKB produced data and drafting the manuscript, ACM and YM contributed to the designing of the study as well as analysis and interpretation of the data, NPB and NCC worked for study design and drafting of the manuscript. All authors contributed to the final editing and approval of the manuscript.
